# Integration of 3D-printed simulation models into a PAG fellowship curriculum: impact on trainee confidence and curriculum acceptability

**DOI:** 10.3389/fmed.2026.1724979

**Published:** 2026-03-31

**Authors:** T. Dumont, A. From, N. P. Rosca, G. Posner, N. Mitsakakis, L. Hayawi, H. O’Reilly, A. T. Lobos, L. McLean

**Affiliations:** 1 CHEO Research Institute, Ottawa, ON, Canada; 2 Division of Gynecology, Department of Surgery, CHEO, Ottawa, ON, Canada; 3 Department of Obstetrics and Gynecology, Faculty of Medicine, University of Ottawa, Ottawa, ON, Canada; 4 Division of General Obstetrics and Gynecology, Department of Obstetrics, Gynecology and Newborn Care, The Ottawa Hospital, Ottawa, ON, Canada; 5 Ottawa Hospital Research Institute, Ottawa, ON, Canada; 6 Department of Mechanical Engineering, University of Ottawa, Ottawa, ON, Canada; 7 Department of Anesthesia, CHEO, Ottawa, ON, Canada; 8 Department of Anesthesiology, Faculty of Medicine, University of Ottawa, Ottawa, ON, Canada; 9 Division of Critical Care, Department of Pediatrics, CHEO, Ottawa, ON, Canada; 10 School of Rehabilitation Sciences, University of Ottawa, Ottawa, ON, Canada

**Keywords:** 3D-printed, fellowship curriculum, pediatric and adolescent gynecology, silicone-casted, surgical simulation, task-trainers

## Abstract

**Introduction:**

Transverse vaginal septum (TVS) is a rare congenital anomaly that poses significant challenges in pediatric and adolescent gynecology (PAG) training due to its low prevalence and surgical complexity. Most PAG fellows may never encounter a case during their fellowship, underscoring the need for high-fidelity simulation tools to enhance procedural readiness.

**Methods:**

Through 3D printing and silicone elastomers, a surgical task trainer replicating TVS anatomy was developed and integrated into a structured simulation curriculum using the Y-plasty technique. The curriculum was delivered during a PAG fellows’ bootcamp and included a didactic session followed by hands-on simulation. Fellows completed pre- and post-session surveys assessing self-perceived confidence and curriculum acceptability, including net promoter score (NPS). Statistical analysis included Wilcoxon signed rank tests and descriptive statistics.

**Results:**

Thirty-six fellows participated, with 66.7% reporting no prior experience with Y-plasty. Post-simulation, median confidence scores increased significantly from 2 (“slightly confident”) to 3 (“moderately confident”) out of 5 (*p* < 0.001). The curriculum received an NPS of 51.5%, indicating strong satisfaction and dissemination potential. Participants estimated that four simulation sessions would be sufficient to achieve self-perceived confidence.

**Discussion:**

The study demonstrates that a single session using a 3D-printed and silicone-cast simulation model was associated with increased self-perceived confidence. The model was well-received and offers a scalable, efficient solution to address educational gaps in PAG fellowship surgical training. Future directions focus on expanding the curriculum to include other vaginal septum types and improving current model performance.

## 1 Introduction

Transverse vaginal septum (TVS) is a rare congenital anomaly resulting from incomplete fusion or failed canalization between the Müllerian-derived upper vagina and the urogenital sinus-derived lower vagina (1). This embryological process typically occurs between the sixth week of gestation and the fifth month, during which the paired Müllerian ducts fuse and canalize to form the uterus, cervix, and upper two-thirds of the vagina. Disruptions in this process can lead to the formation of a transverse septum, which may present clinically in pubertal patients with primary amenorrhea, cyclical abdominal pain, and, in severe cases, urinary retention and increased abdominal girth.

TVS is estimated to occur in approximately 1 in 30,000 females, making it a particularly rare condition (2). As a result, pediatric and adolescent gynecology (PAG) fellows, whose training spans only one to two years, may never encounter a case during their fellowship, let alone perform the surgical correction. A PAG fellow is a physician who has completed a residency in obstetrics and gynecology and is undergoing specialized training in the gynecologic care of children and adolescents, typically from birth to age 18–21. The surgical management of TVS is technically demanding due to the variability in septum location and thickness, and the potential for complications such as vaginal stenosis. Multiple surgical approaches exist, but there is no standardized method, and no simulation tools have been developed to support training in this area.

**Figure 1:**
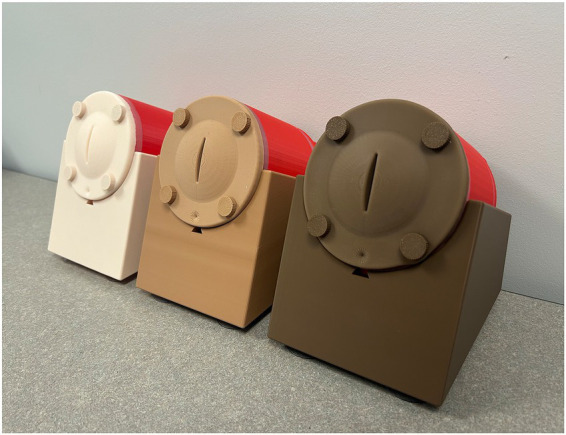
Transverse vaginal septum models developed by our team (external view with the three skin tones).

**Figure 2:**
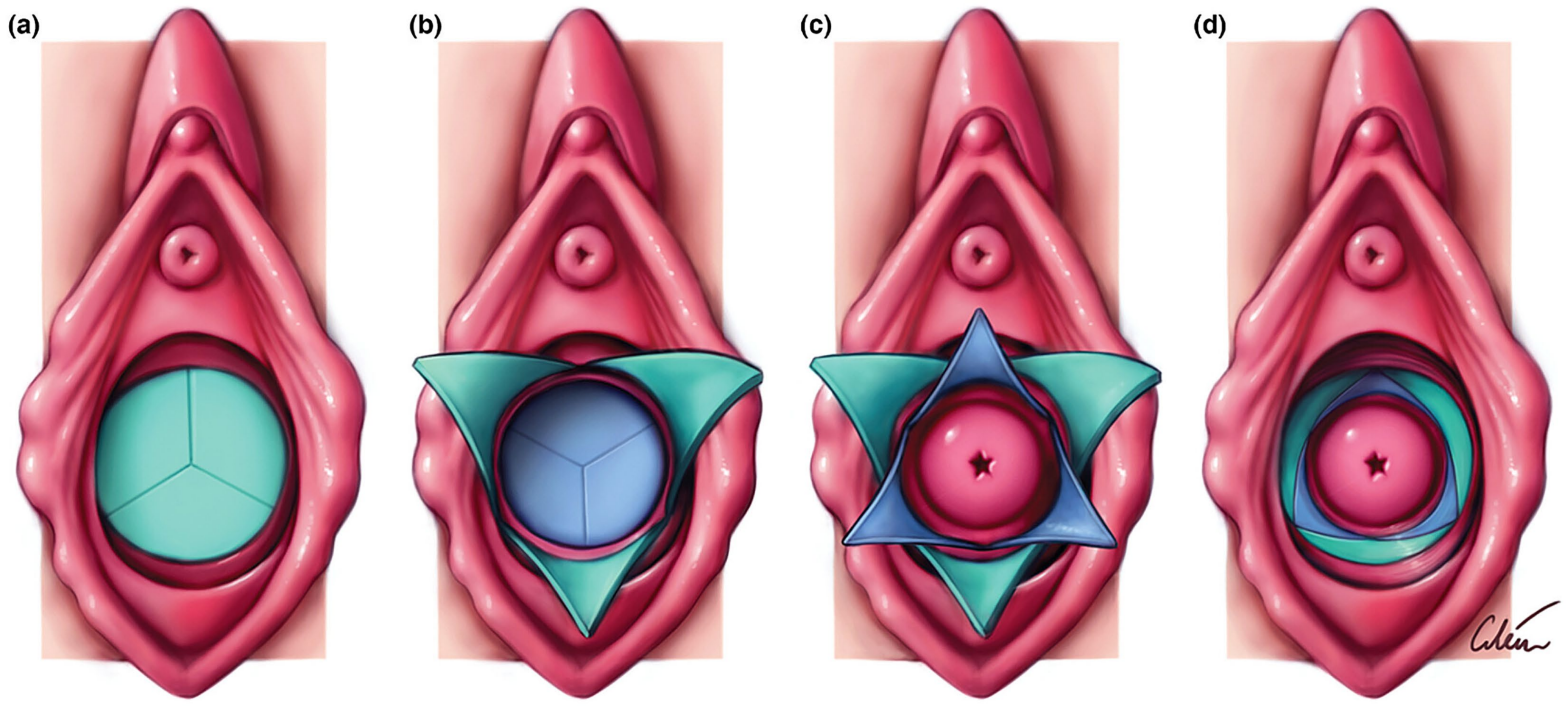
Schematic diagram of vulva and vaginal canal before **(a)** and after **(d)** release of septum with interdigitating Y-plasty technique. **(a)** Imperforate vaginal septum; note external and internal lamellae. Interstitial areolar tissue of variable thickness normally occupies interlamellar space. **(b)** Inverted Y-plasty on external lamella produces three external flaps; these will eventually be turned inwards. **(c)** Y-plasty on internal lamella produces three internal flaps; these are turned outwards. **(d)** External flaps are inverted and interdigitated with internal ones. Note zigzag pattern of resulting scar. Reproduced with permission from John Wiley and Sons (4).

Given these challenges, there is a critical need for innovative educational tools that can provide realistic, hands-on experience in a controlled environment. Simulation-based education, particularly using anatomically accurate 3D-printed and silicone-cast models, offers a way to support exposure and increase self-perceived confidence, though it does not replace assessment of real-world operative skills. Our team bridged this gap by developing a high-fidelity 3D-printed model for TVS resection using the Y-plasty technique (Figure 1). Y-plasty is a reconstructive surgical technique for transverse vaginal septa in which opposing Y-shaped incisions are made in the septal lamellae and the resulting flaps are interdigitated, creating a zig-zag scar without excising tissue (Figure 2) (3, 4). This approach preserves vaginal length and caliber while altering scar geometry to reduce circumferential contracture, thereby lowering the risk of postoperative vaginal stenosis compared with simple excision or cruciate incisions. As shown in both articles, Y-plasty is particularly advantageous for thin septa and in adolescents, offering reliable patency with fewer complications and less need for prolonged dilation (3, 4). The aim of this paper is to review the integration of this model into a structured simulation curriculum and to evaluate its impact on trainees’ self-perceived confidence and curriculum acceptability.

## 2 Materials and methods

### 2.1 Study setting and participants

The study was designed as a curriculum development project with an embedded evaluation component. It was conducted during a fellows’ bootcamp held at an international meeting, the North American Society for Pediatric and Adolescent Gynecology’s (NASPAG) Annual Clinical and Research Meeting (ACRM) in Toronto, Canada in April 2025. Participants included both incoming and current fellows in pediatric and adolescent gynecology. The intervention consisted of a one-hour simulation-based session utilizing a 3D-printed surgical task trainer specifically designed to replicate the anatomical features of a transverse vaginal septum. The curriculum started with a short didactic presentation to review the embryology, symptom presentation, and treatment options of transverse vaginal septa. Fellows were then grouped in pairs of two and were given a 3D-printed surgical task trainer with the necessary equipment and sutures to perform the excision of the vaginal septum using the Y-plasty technique. The course instructor, TD, then led the fellows, step by step, through the surgical procedure while over half-dozen PAG providers with surgical expertise in Mullerian anomalies, who were attending the international meeting, were circulating around the room and coaching the fellows through the surgical steps.

### 2.2 Survey and outcome measures

At the start of the one-hour session, participants provided consent and completed the pre-curriculum survey via an online survey. Study data were collected and managed using REDCap electronic data capture tools hosted at the CHEO RI (5, 6). Post-curriculum, the 2nd online survey was completed with RedCap. The primary outcome was the fellows’ self-perceived ability to independently perform the surgical procedure before and after the completion of the curriculum using a 5 point-Likert scale (1-not confident, 5-very confident; score created with the authoring team). The secondary outcomes included: the likelihood that the fellows would recommend the surgical simulation curriculum to other fellows or fellowship program directors using the net promoter score (NPS) (7, 8). NPS scores were between 0 (not at all likely) to 10 (extremely likely). Additionally, we assessed how accurately these 3D-printed simulation models reflected reality in the three areas: anatomy, operability, and tissue handling using a 5 point-Likert scale (1-not accurate, 5-very accurate).

### 2.3 Statistical analysis

Descriptive statistics to summarize participant responses used frequencies and percentages for categorical variables, and medians and quartiles for continuous variables. To assess the change in pre- and post-session confidence score in performing Y-plasty, we used a Wilcoxon signed rank test for paired data. The NPS percentage difference was calculated by taking the percentage of participants who responded with nine or ten (promoters) and subtracting the percentage who responded with zero through six (detractors). A score above 20% is considered indicative of strong dissemination potential (7–9). We presented the percentages of responses to Likert scale questions assessing the accuracy of the simulation models to reflect reality in the three previously listed areas using graphical presentation.

## 3 Results

**Figure 3:**
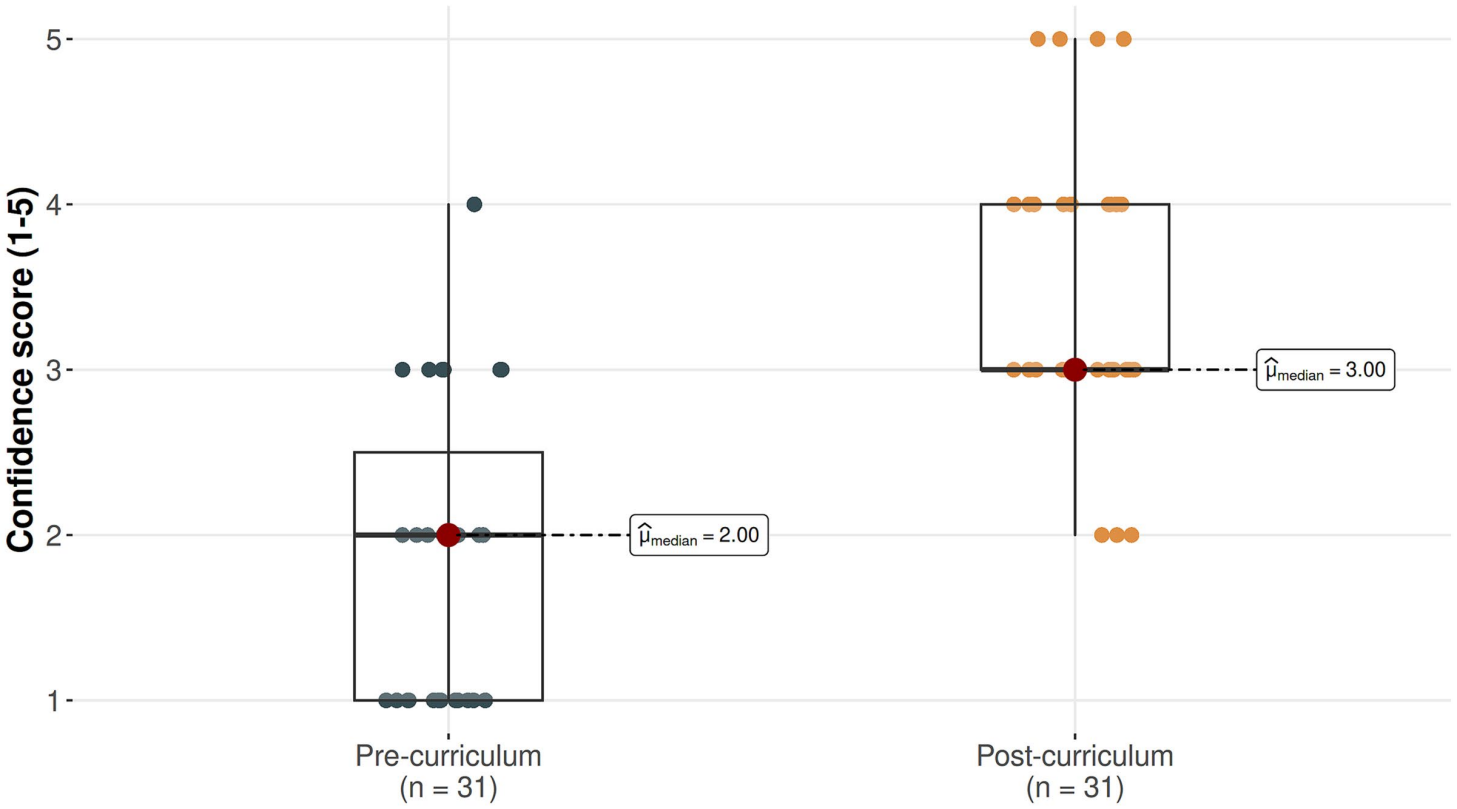
Confidence score for transverse vaginal septum resection presented as a continuous scale for paired data. *N* = 31. Higher values indicate more confidence (1 = not confident, 5 = very confident).

A total of 36 fellows participated in the simulation session. Of these, 24 (66.7%) reported having never performed a Y-plasty prior to the session (Table 1). Following the simulation, there was a statistically significant improvement in median confidence scores, which increased from 2 out of 5 (“slightly confident”) to 3 out of 5 (“moderately confident”), with a *p*-value of less than 0.001 (Figure 3). The simulation session improved self-reported confidence; however, self-confidence does not indicate technical skill nor independently verified competence.

**Table 1:** Characteristics of the participants (*N* = 36).

Variable	Value
Year of fellowship currently in	
Incoming fellow	10 (27.8%)
First year	15 (41.7%)
Second year	7 (19.4%)
Third year	4 (11.1%)
Missing	2
Standard duration of the fellow’s university’s PAG fellowship	
1 year	5 (13.9%)
2 years	29 (80.6%)
3 years	2 (5.6%)
Missing	2
Location of the current fellowship	
Canada	6 (16.7%)
USA	28 (77.8%)
Other country	2 (5.6%)
Missing	2
How many times the fellow was the primary surgeon of longitudinal vaginal septum resection	
0	9 (25.0%)
1–2	19 (52.8%)
3–4	6 (16.7%)
5–10	1 (2.8%)
Greater than 10	1 (2.8%)
Missing	2
How many times the fellow was the primary surgeon of oblique vaginal septum resection	
0	22 (61.1%)
1–2	11 (30.6%)
3–4	3 (8.3%)
5–10	0 (0.0%)
Greater than 10	0 (0.0%)
Missing	2
How many times the fellow was the primary surgeon of transverse vaginal septum resection	
0	24 (66.7%)
1–2	11 (30.6%)
3–4	0 (0.0%)
5–10	1 (2.8%)
Greater than 10	0 (0.0%)
Missing	2

Table 2 shows that the curriculum received an NPS of 51.5%, reflecting a high level of satisfaction and a strong likelihood of recommendation to peers and program directors. Participants were asked “Would repeating these simulations multiple times increase your ability to perform the surgical procedures independently?” and 2/33 (6.1%) did not feel the need for any additional sessions whereas 22/33 (66.7%) and 9/33 (27.3%) felt that 1–2 sessions and 3–4 sessions, respectively, would be needed. Therefore, a maximum of four additional simulation sessions would be required to achieve procedural competency according to participants. Although additional sessions might further improve self-perceived confidence, these subjective estimates do not reflect objective measures of operative ability. Ninety-seven percent of the fellows felt that this curriculum should be repeated yearly during the fellows’ bootcamp. These findings underscore the model’s potential as a valuable tool in surgical education.

**Table 2:** Percentage difference in NPS scores of those who scored 9, 10 (promoters) and those who scored ≤6 (detractors).

NPS scores (9, 10); *n* (%)	NPS scores (0 to 6); *n* (%)	Difference between %
22/33 (66.7%)	5/33 (15.2%)	51.5%

**Figure 4:**
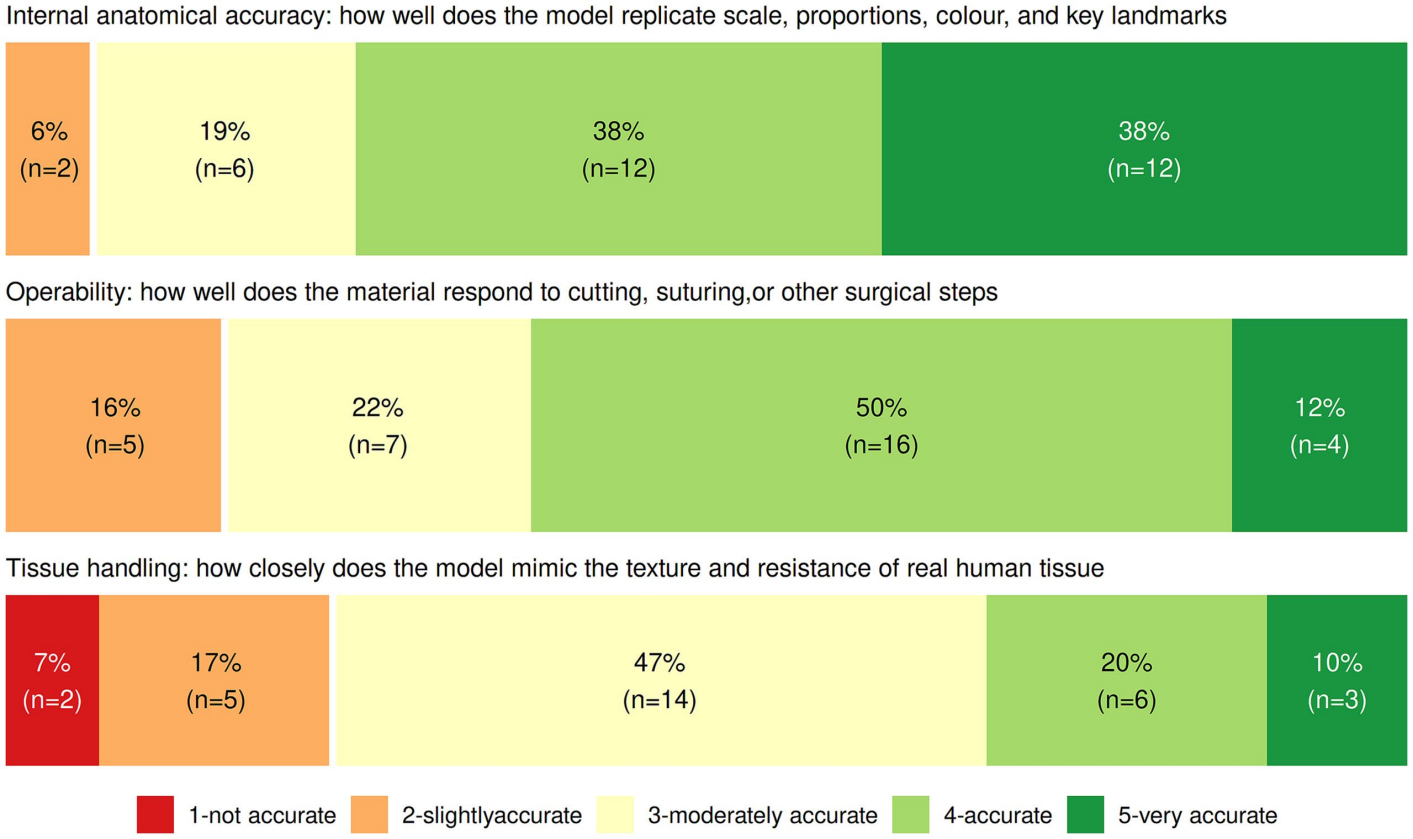
Participants’ ratings of how accurately the 3D-printed simulation models reflected reality in the three domains: anatomy, operability, and tissue handling (1-not accurate, 2-slightly accurate, 3-moderately accurate, 4-accurate, 5-very accurate).

Figure 4 shows that 76% of the participants reported that the simulation models were accurate or very accurate in replicating the anatomical size, proportions and colors of a real tissue. Sixty two percent thought that the models were accurate or very accurate in terms of operability defined by responsiveness to surgical manipulation such as cutting and suturing. However, only 30% of participants reported that the models were accurate or very accurate in mimicking the texture and resistance of a real tissue. This highlights a limitation in realism that might constrain any assumption of skill transfer.

## 4 Discussion

This study demonstrates that a 3D-printed simulation model can significantly improve trainee confidence in performing a rare but complex surgical procedure – Y-plasty for TVS resection. Given the low prevalence of congenital Müllerian anomalies such as TVS, many PAG fellows will not encounter this pathology during their training. Our simulation model, integrated within a structured curriculum, addresses this critical educational gap and provides a promising tool for enhancing procedural readiness. The details of how this model was created will be published in an engineering journal.

The statistically significant increase in fellows’ self-reported confidence after a single session is particularly noteworthy. While confidence is not a direct measure of competence, it is an important component of surgical preparedness, especially for technically demanding procedures that require anatomical precision and decision-making under uncertainty. For trainees with little or no prior exposure to TVS resection, the opportunity to visualize, practice, and receive immediate feedback in a risk-free setting is invaluable. Our findings indicate a measurable change in self-perceived confidence only; no evidence regarding skill acquisition nor operative performance is provided with this study.

Equally important is the model’s strong reception, reflected in a NPS of 51.5%. This suggests acceptability and enthusiasm for the training model and curriculum. Participants believed that as few as four additional sessions might improve self-perceived confidence, but such perceptions cannot be extrapolated to procedural competency from this study. These results align with broader trends in surgical education, where high-fidelity simulation is increasingly recognized as a powerful tool for teaching infrequent but high-risk procedures.

This curriculum was delivered during a professional conference in a traditional classroom setting, which introduces several important limitations. Space constraints, time limitations inherent to conference schedules, and variability in available surgical instrumentation may have limited opportunities for prolonged hands-on practice or individual repetition of the task. Faculty-to-learner ratios, while favorable compared to most clinical environments, were nevertheless constrained by simultaneous conference commitments, potentially affecting the consistency of real-time feedback across learners. Additionally, classroom-based simulation lacks some elements of the operating room environment, including full ergonomic positioning, appropriate lighting, active blood loss, anesthesia workflow, and perioperative decision-making under clinical pressure. These factors may limit the direct transferability of self-perceived confidence gains to immediate operative performance. Despite these challenges, delivering the curriculum in this setting allowed exposure of a geographically diverse cohort of fellows to a rare surgical procedure that many may otherwise never encounter, underscoring the feasibility and educational value of conference-based simulation for low-frequency, high-complexity surgeries. Future iterations of this curriculum could benefit from longitudinal, institution-based simulation sessions that allow repeated practice, standardized equipment, and objective performance assessment.

Our study also builds on prior needs assessments from NASPAG fellowship program directors (unpublished survey) that indicated a need to develop educational tools specifically for congenital gynecologic anomalies. To our knowledge, this is the first reported use of a 3D-printed model specifically designed for Y-plasty in TVS resection. By simulating the variable location and tissue characteristics of a septum, our model supports individualized learning and offers opportunities to rehearse surgical steps that are otherwise difficult to teach. The model’s success in a real-world bootcamp setting, with a geographically and professionally diverse cohort of fellows as seen in Table 1, underscores its practical value.

This work is not without limitations. First, confidence ratings are subjective, and further research is needed to assess skill acquisition using objective structured assessments or expert ratings. Second, while the model was piloted in a single educational setting, future studies should evaluate its reproducibility, durability, and educational impact across multiple sites including low-resource setting outside of North America. Additionally, we recognize that real-life surgical performance involves intraoperative decision-making, hemostasis, and soft tissue management, all of which cannot be fully replicated in a simulation model. However, by familiarizing trainees with anatomy, instrumentation, and core procedural steps, this model can increase self-perceived confidence but cannot be assumed to improve operative performance without objective skills assessment.

Finally, the positive response to this initial prototype encourages further development. Plans are underway to (1) refine the model’s realism, especially texture and resilience of the tissue given this was the lowest scored domain (Figure 4), (2) incorporate a broader range of congenital vaginal anomalies, including longitudinal and oblique septa, and (3) ensure its external validity by testing these models in fellowship centers across the world with varied surgical instruments and experts. We also envision disseminating these models and curricular tools to PAG fellowship programs internationally to support equitable access to training resources as well as to other training programs (pediatric surgery/urology, minimally invasive gynecology, urogynecology, reproductive endocrinology and infertility) that may perform these surgeries where no PAG providers exist.

## 5 Conclusion

A single session using a 3D-printed simulation model significantly increased fellows’ self-perceived confidence in performing Y-plasty for TVS resection. The high net promoter score further supports the curriculum’s value and its potential for widespread adoption. Future directions include improving model realism, validating objective performance metrics, and evaluating whether repeated use influences both confidence and skill acquisition. Furthermore, creating and incorporating simulation models for longitudinal and oblique vaginal septum resections would enable the development of a comprehensive suite of vaginal septum models for fellowship-level surgical training.

## Data Availability

The raw data supporting the conclusions of this article will be made available by the authors, without undue reservation. Researchers outside of this specific study may request access to the coded data for new research purposes. Participants will not be asked to provide additional informed consent for the use of the coded data for future research.
